# Randomized trial of DRV/r or LPV/r QD monotherapy vs maintaining a PI/r-based antiretroviral regimen in persons with suppressed HIV replication

**DOI:** 10.7448/IAS.17.4.19809

**Published:** 2014-11-02

**Authors:** Carmela Pinnetti, Patrizia Lorenzini, Alessandro Cozzi-Lepri, Ottou Sandrine, Chiara Tommasi, Mauro Zaccarelli, Carlo Federico Perno, Maria Rosaria Capobianchi, Enrico Girardi, Andrea Antinori, Adriana Ammassari

**Affiliations:** 1 Clinical Department, National Institute for Infectious Diseases Lazzaro Spallanzani IRCCS, Roma, Italy; 2 Research Department of Infection and Population Health, University College, London, UK; 3 Virology Department, University of Tor Vergata, Roma, Italy; 4 Virology Department, National Institute for Infectious Diseases Lazzaro Spallanzani IRCCS, Roma, Italy; 5 Epidemiologic Department, National Institute for Infectious Diseases Lazzaro Spallanzani IRCCS, Roma, Italy

## Abstract

**Introduction:**

PI/r monotherapy has been suggested as an attainable maintenance strategy in patients achieving stable HIV suppression in plasma. The objective of trial was to compare the virological outcome of two different PI/r QD monotherapy strategies (LPV/r or DRV/r) with maintaining a triple PI/r-based ARV regimen.

**Material and Methods:**

Phase III, open-label, non-inferiority (−12% margin), randomized trial of HIV adults with HIV-RNA <50 cp/mL for at least 48 weeks while on PI/r-based cART, CD4 nadir >100 cell/mm^3^, without previous PIs virological failure. Eligible patients were randomized to continue PI/r+2NRTIs (Arm A), to switch to LPV/r 800/200 mg QD monotherapy (Arm B), or to switch to DRV/r 800/100 mg QD monotherapy (Arm C). Primary endpoint was proportion of patients with plasma HIV-1 RNA <50 cp/mL (TLOVR) at 48w by intent to treat (ITT) analysis (missing/re-induction=failure). FDA snapshot and ITT switch-included analysis (ITT-SI) were also used. In ITT-SI, patients who had <50 copies/mL at 96w were counted as successes even if they had confirmed HIV-RNA elevations and had subsequently successfully intensified by NRTI.

**Results:**

Due to slow recruitment, only 103 patients were included. No differences were observed between the three arms with respect to gender, age, HIV transmission, CD4 nadir and at screening. At randomization, 61 patients were receiving TDF/FTC (60%), 19 ZDV/3TC (18%), 8 ABV/3TC (8%), 75 LPV/r (73%), 13 ATV/r (13%), 4 DRV/r (4%). Differences in proportion of virological success by groups using Arm A as comparator according to FDA TLOVR were reported in Figure 1. Similar results were obtained by Snapshot analysis. Of 14 patients with virological failure, 8 patients restarted triple therapy with 2NRTI and 7/8 regained a VL <50 cp/mL over time. According to ITT-SI analysis, 96 week differences [95% CI] were −5.7 [−29.6; +18.2] in Arm B, and +19.6 [−1.6; +40.8] in Arm C. A GRT was performed in 6/14 patients (one not amplifiable; four without mutations; one showed E138A).

**Conclusions:**

Compared to maintaining a PI/r-based triple ARV regimen, LPV/r QD monotherapy tended to have higher rate of virological failure and of discontinuation due to adverse event. In contrast, the response rate at week 96 during DRV/r QD mono-therapy was non-inferior to that of triple PI/r-based ARV therapy. A re-induction with 2NRTI was adequate to obtain an undetectable viremia in most of patients with virological failure.

**Figure 1:**
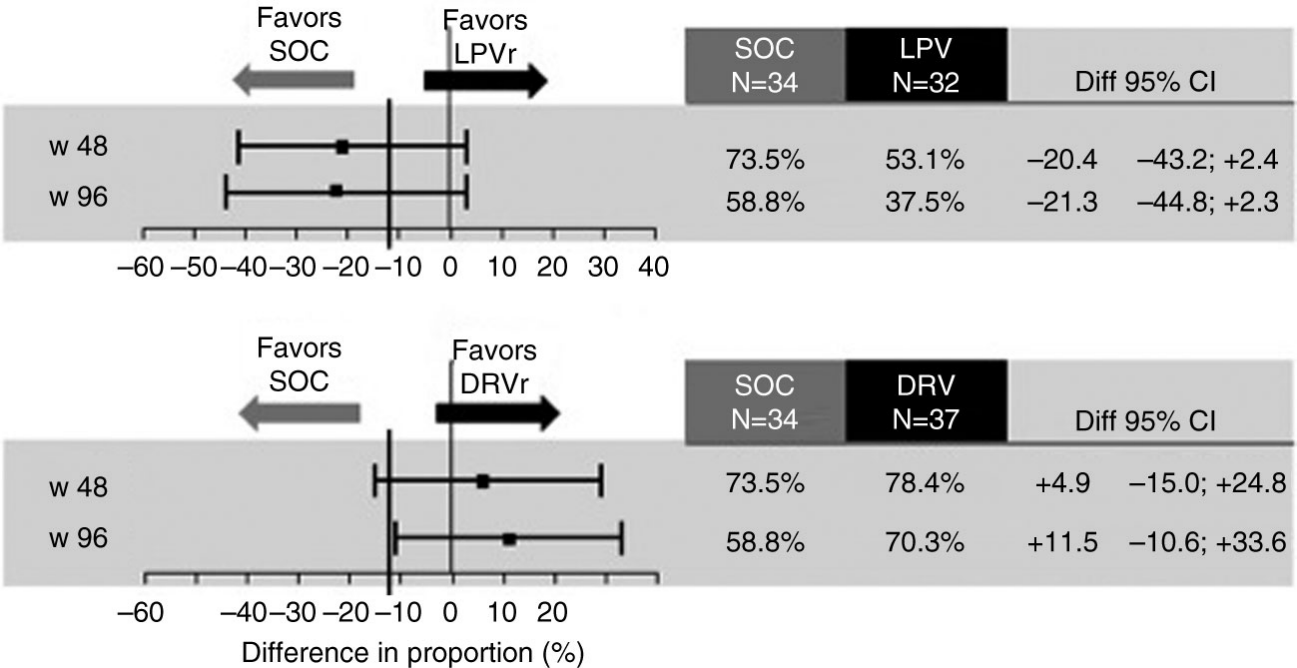
Primary end point, ITT analysis according with TLOVR algorithm (missing/re-induction=failure).

